# A home-based tele-rehabilitation exercise system for patients after knee replacement surgery

**DOI:** 10.1186/s12891-024-07731-4

**Published:** 2024-07-31

**Authors:** Farnaz Salehian, Zahra Mahmoudzadeh-Sagheb, Amin Kordi Yoosefinejad, Somayyeh Zakerabasali

**Affiliations:** 1 Student Research Committee, Department of Health Information Management, School of Health Management and Information Sciences, Shiraz University of Medical Sciences, Shiraz, Iran; 2 Department of Health Information Management, Health Human Resources Research Center, School of Health Management and Information Sciences, Shiraz University of Medical Sciences, Shiraz, Iran; 3 Department of Physical Therapy, School of Rehabilitation Sciences, Rehabilitation Sciences Research Center, Shiraz University of Medical Sciences, Shiraz University of Medical Sciences, Shiraz, Shiraz, Iran; 4 Clinical Education Research Center, Health Human Resources Research Center, Department of Health Information Management, School of Health Management and Information Sciences, Shiraz University of Medical Sciences, Shiraz, Iran

**Keywords:** Telerehabilitation, Total knee replacement, TKR, Usability, Prototype, System, Usability, Knee joint replacement, TKR

## Abstract

**Background:**

Knee arthritis is a destructive disorder that affects the knee joints and causes pain and reduced mobility. Drug treatments, weight loss, and exercise control the symptoms of the disease, but these methods only delay the disease process and eventually, knee joint replacement surgery will be needed. After surgery, with the help of a proper physiotherapy program, full recovery takes an average of 6–12 months. However, currently, there is no similar tool to facilitate this process in Iran.

**Objective:**

The purpose of this research is to design and develop the prototype of a rehabilitation system for patients after knee replacement surgery, which provides patients with information and appropriate physiotherapy programs.

**Methods:**

This study was development-applied and was done in three stages. In the first stage, the needs and content of education and therapeutic exercises were prepared in the form of a checklist, and then the necessity of each item was checked in the evaluation sessions. In the second stage, the prototype of the system was developed using Adobe XD software and based on the requirements approved from the previous stage. In the third stage, the usability of the program was analyzed from the point of view of experts using the exploratory evaluation method with Nielsen’s 13 principles of usability.

**Results:**

At first, the system requirements were extracted and prepared in two checklists of content (51 exercises) and capabilities (60 items). Then after a survey of experts based on the Delphi technique, content information (43 exercises) and functional and non-functional requirements (53 items) were obtained. A survey of experts helped to finalize the information elements, categorize them, and prepare the final version of the checklists. Based on this, the system requirements were classified into 11 categories, and the training content was classified into 3 training periods. Finally, the design and development of the system was done. This system has admin, physician, and patient user interfaces. The result of usability showed that this system is efficient and there are only a few problems in the feature of helping users to detect and recover from errors.

**Conclusion:**

It seems necessary to develop a system based on the principles of usability by therapists and rehabilitation specialists to train and monitor the remote rehabilitation process of patients after knee joint replacement at home. And the importance of involving stakeholders in the design and development of remote rehabilitation systems is not hidden from anyone. Kara system has all of the above.

**Supplementary Information:**

The online version contains supplementary material available at 10.1186/s12891-024-07731-4.

## Background

With the increase in the incidence of knee arthritis, which occurs due to factors such as population aging, obesity, and joint damage, the need for knee joint replacement surgery has increased. Also, with increasing expectations for continuing a physically active lifestyle and increasing demand for surgery at younger ages, the importance of this surgery is felt more these days [1–3].

In the last decade, the method of total knee replacement with faster recovery has been implemented in many hospitals. This method, by using several models of painkillers, timely action, and fluid management [4, 5], leads to a significant reduction in the duration of hospitalization of patients after total knee replacement, in which patients do not need to stay overnight in the hospital [6]. However, full recovery after this surgery takes 6–12 months on average if an appropriate physiotherapy program is received [7].

Due to the short stay of patients in the hospital, self-management of patients has become an important factor in improving their health outcomes. In the case of total knee replacement surgery, self-management after the operation usually starts with controlling the pain level, followed by physical therapy exercises and daily self-care activities [8–11]. Patient education should also be provided to prepare patients to better manage their new condition when they return home.

Today, with technological progress and new achievements in information and communication technology, we can witness fundamental changes in the way people access facilities and services. One of these new technologies is Tele-rehabilitation which helps patients self-manage. Using Tele-rehabilitation as an alternative to face-to-face visits has many benefits for patients and therapists. For example, reducing the time and distance costs of commuting, reducing the possibility of contracting infectious diseases, especially in the conditions of the coronavirus pandemic [12], or easier access to people living in remote and rural areas are some of the benefits of Tele-rehabilitation for patients [13].

However, currently, there is no Tele-rehabilitation tool to facilitate physiotherapy after total knee replacement in Iran. The purpose of this research is to design and develop such system that allows patients to access information, perform exercises, and communicate.

## Methods

### Study design

An expert-based design is carried out after several meetings with the research team and conducting the necessary investigations, the system prototype was developed based on the approved requirements. The system prototype was built during these steps: requirements analysis and identification, requirements validation, design/prototyping, and evaluation.

### Step 1: identification of application requirements

This step includes reviewing past studies and analyzing similar applications to determine the requirements (capabilities and content) of the application. Also, to collect the content, the guidelines and reference books related to this disease were selected and studied under the supervision of the relevant specialist to prepare the necessary content for the software.

We conducted this review by searching the online databases of PubMed, Web of Science, and Scopus for the relevant literature using keywords and operational phrases. The search was conducted between January 2017 and April 2022. The keywords and search strategy are described in Table 1.

The studies were retrieved and the duplicate records were removed. We followed a two-step screening process to sort the relevant results. First, we examined the title and abstract of the records, and the irrelevant articles were removed. Then, their full texts were evaluated based on their cohesion to the inclusion/exclusion criteria, and the relevant ones were included.

In step two, articles that met the following conditions were reviewed: (A) Studies on remote rehabilitation after knee replacement surgery, (b) studies that had a health application intervention on telerehabilitation after knee replacement surgery.

We read the full text of selected articles. Then, based on the full texts, the articles were classified into 3 categories: 1-Articles that talked about the application but did not talk about the capabilities of that application. 2-Articles that talked about some of the capabilities and features of the application, but did not talk about all the features of the application. 3-Articles that fully discuss all the features of the application.

Finally, the articles that were placed in the third category were selected as final articles and were analyzed to extract the capabilities of their applications.

After extracting operational requirements from the literature review and similar applications and preparing a checklist of capabilities, 5 patients who recently underwent knee replacement surgery were surveyed about the needs of the app.

**Table 1:** Keywords and search strategy

**keywords**	mobile health, mhealth, m-health, Smartphone, Mobile App*, total knee arthroplasty, TKA, total knee replacement, TKR, Knee Replacements, Knee Arthroplasty
**search strategy**	[(mobile health) OR (mhealth) OR (m-health) OR (Smartphone) OR (Mobile App*)] **AND** [(total knee arthroplasty) OR (TKA) OR (total knee replacement) OR (TKR) OR (Knee Replacements) OR (Knee Arthroplasty)

### Step 2: validation of application requirements

With the results of Step 1, a list of capabilities and list of suggested contents were prepared in the form of a content checklist and a capabilities checklist. The content checklist was distributed among 10 physiotherapy specialists and the capabilities checklist was distributed among 12 experts from the fields of physiotherapy, health information management, and medical informatics and they were asked to comment on the necessity of each item and requirement.

Based on the focus group meeting with the research team, and classification of items, finally we reached the capability checklist, including 60 components in 11 sections: patient demographic data, educational-practice module capabilities, capabilities of social support, capabilities of increasing motivation, capabilities of patient monitoring, reminder capabilities, Communication capability, system security capability, ability to get reports, capabilities related to the user interface and the ability to ask questions in the program.

The content checklist contains a list of necessary training and exercises for patients who use this application. Exercises are divided into three periods according to the reference book (Therapeutic Exercise: Foundations and Techniques, by Kisner [14]) and with the approbation of the physiotherapist: the initial phase of rehabilitation (for 4 weeks), the middle phase (protection/movement phase) of rehabilitation from The fifth week after surgery (for 8 to 12 weeks) and the last stage of rehabilitation (minimal protection/return to function), there are 51 exercises in the content checklist.

To confirm the necessity of items in the checklists, we used the Delphi technique, and both checklists were designed based on a 5-point Likert scale: I strongly agree: 5, I agree: 4, I have no opinion: 3, I disagree: 2, I strongly disagree: 1, and finally a list of functional capabilities and suggested contents which was agreed by the majority (above 75% of the experts) was included in the design of the system. For majority agreement, each item must receive at least an average score of 3.75 out of 5 in the survey.

### Step 3: interface design and prototyping

After several meetings with the research team and conducting the necessary investigations, the system prototype was developed based on the approved requirements.

This system has admin, physiotherapist, and patient user interfaces. The admin user interface is web-based and designed by using Adobe XD software. The admin of the system is able to upload the content of the application, and register the care team and patients through the web application interface. We chose the name of the application as Knee Arthroplasty Rehabilitation Application (KARA).

The patient and care team user interface is based on Android and is also designed using Adobe XD software. The care team can track the patient’s condition and view the reports sent by the patient, communicate with the patient for consultation, determine the therapeutic personalized exercise program for patients, and provide the necessary instructions in the form of video for each exercise through their user interface. Communication, education, reporting, information, and question-and-answer services are provided for patients in this application. Through the patient user interface, patients can submit information (demographic and clinical), view educational content and physical therapy exercises, and give feedback to the care team by completing the knee injury and arthritis outcome (KOOS) questionnaire when the physiotherapist asks.

Also, we used MAGIC POSER software to create video and image content of trainings and exercises, and we converted the output images from this software into video files with photo to GIF converter software.

### Step 4: system evaluation

At this stage, the applicability of the prototype was evaluated with the help of experts using the Heuristic evaluation method.

This method was developed Based on the principles determined by Nielsen, it is used to identify potential system applicability problems by 3 evaluators, and a number is assigned to the system as a degree of severity (from the absence of problems to the presence of catastrophic problems) [15].

To evaluate the applicability, Nielsen’s principles in the form of a checklist were provided to 3 evaluators who were selected by the research team. The evaluators studied in the field of computer engineering or medical informatics and were familiar with the Heuristic evaluation [15, 16].

First, the app was sent to the evaluators and they were asked to evaluate the app, then a shortened version of Nielsen’s usability checklist, whose validity and reliability were measured and has 13 principles and 54 questions [16] and it was given to the evaluators and the evaluators answered the questions. They evaluated the app independently and checked the compatibility of the user interface with each of the Heuristic principles separately.

## Results

### Step 1, 2: identification and validation of application requirements

**Fig. 1:**
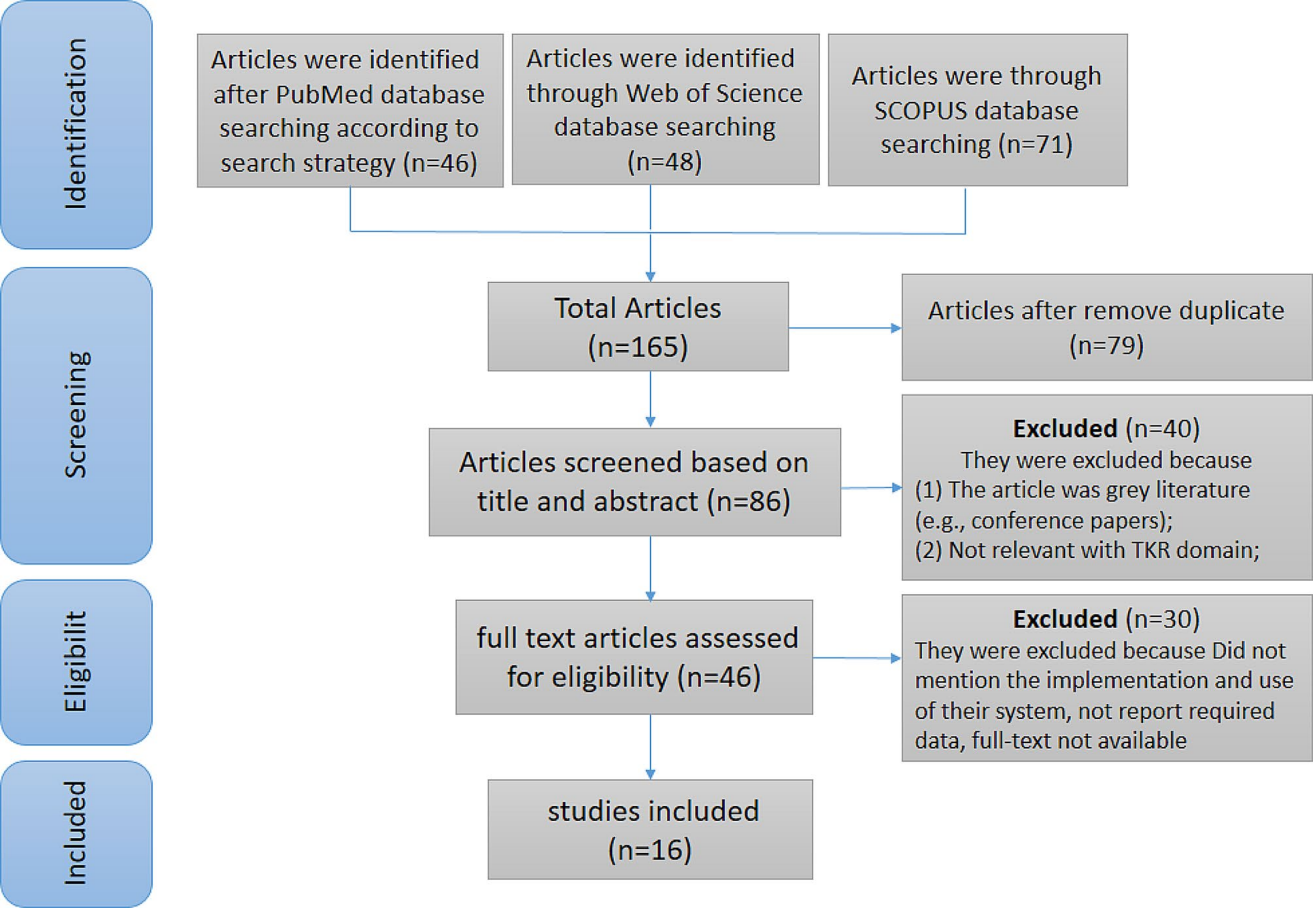
The flow diagram for the identification, screening, and eligibility of studies based on PRISMA

After Reviewing PubMed, Web of Science, SCOPUS, and Medline databases, 165 articles were retrieved, and by removing duplicate articles, we reached 86 articles. Then, by evaluating the title and abstract of the studies, 46 articles were selected for full-text evaluation and 16 of them were included in this study. A flow chart of search results is presented in Fig. 1.

After reviewing the selected articles, the capabilities checklist was prepared. The results of surveys with patients were also added to the items of the checklist, of course, more than 80% of their requests overlapped with the capabilities obtained from the literature review, and the rest of their needs such as questions and answers and frequently asked questions were also added to the initial checklist. Finally, the capabilities checklist had 60 items in 11 sections and included patient demographic data, capabilities of the educational-practice module, social support, increasing motivation, and patient monitoring, the ability to remind and communicate with others, system security, the ability to get reports, the capabilities related to the user interface and the ability to ask questions with the app. results of the experts’ survey showed that 7 items were deleted.

The information elements of race, marital status, spouse’s education and employment status, and the use of alcoholic beverages and tobacco, from the demographic data section of the patient, were among the deleted items. The remaining 53 items are considered in the development of the software. You can see the Capabilities checklist in the attachment.

The summary of the results of the capabilities checklist is shown in Table 2 as a list of functional and non-functional requirements of the application.

The total number of exercise items in the content checklist was 51, of which 8 items were removed after the experts’ survey, and for the remaining 43 items, text and image or video content was prepared and placed in the app database.

**Table 2:** List of functional and non-functional requirements of Kara application

Row	Functional and non-functional requirements	Description	References
1	Collecting information about the user’s social and demographic characteristics	Gender, Age, Race, BMI, level of education, working status, Marital status, Partner’s education, Partner’s employment, Number of pregnancies (for women), current Alcohol use and current Tobacco use.	[17]
2	Teaching exercises to patients in the form of text and video	educate the patients with provide information on the disease, information for preparation to hospital stay, frequent monitoring with the tools, Psychoeducation, relaxation techniques, Yoga exercises guidelines	[17–22]
3	Teaching how to do daily personal activities	given information on rehabilitation, sports, sleep and returning to work, step-by-step tutorial training program	[17, 18, 22, 23]
4	Social support	Viewing frequently asked questions and exchanging opinions in the discussion forum for patients, interaction with other patient with TKA, social activity, support groups	[24]
5	Capabilities to increase motivation in patients	Patients can set goals, interact with the app on a regular basis, congratulates them for special accomplishment	[18, 23, 25–27]
6	Monitoring the patient	evaluating the patient using the KOOS questionnaire, managing body weight and nutrition, follow-up text message, record symptoms and result of training, viewing the patient status report	[17, 18, 20, 22–24, 26–30]
7	Ability to communicate	facilitate communication after discharge, Interaction with therapist, feedback on physical activity, asking questions from expert	[17, 18, 20, 22, 24, 28–31]
8	set a reminder to remind doing exercises	possibility of setting a reminder to remind the patient perform exercises and other activities, especial remind during phases when there is reduced contact with health care Professionals	[17, 20, 22]
9	Privacy and security	Security of patient data, confidentiality of patient data, Authentication	[32–34]
10	Usability and attention to simplicity and beauty	Aesthetic and minimalist design, Flexibility and efficiency of use	[23, 35–37]
11	The ability to prevent and fix errors	Help users recognize, diagnose and recover from errors	[32–34]

### Step 3: interface design and prototyping

According to the requirements extracted from the previous step, the prototype of the program was developed using Adobe XD software. Patient, physiotherapist, and administrator user interfaces are described below.

### Patient user interface

**Fig. 2:**
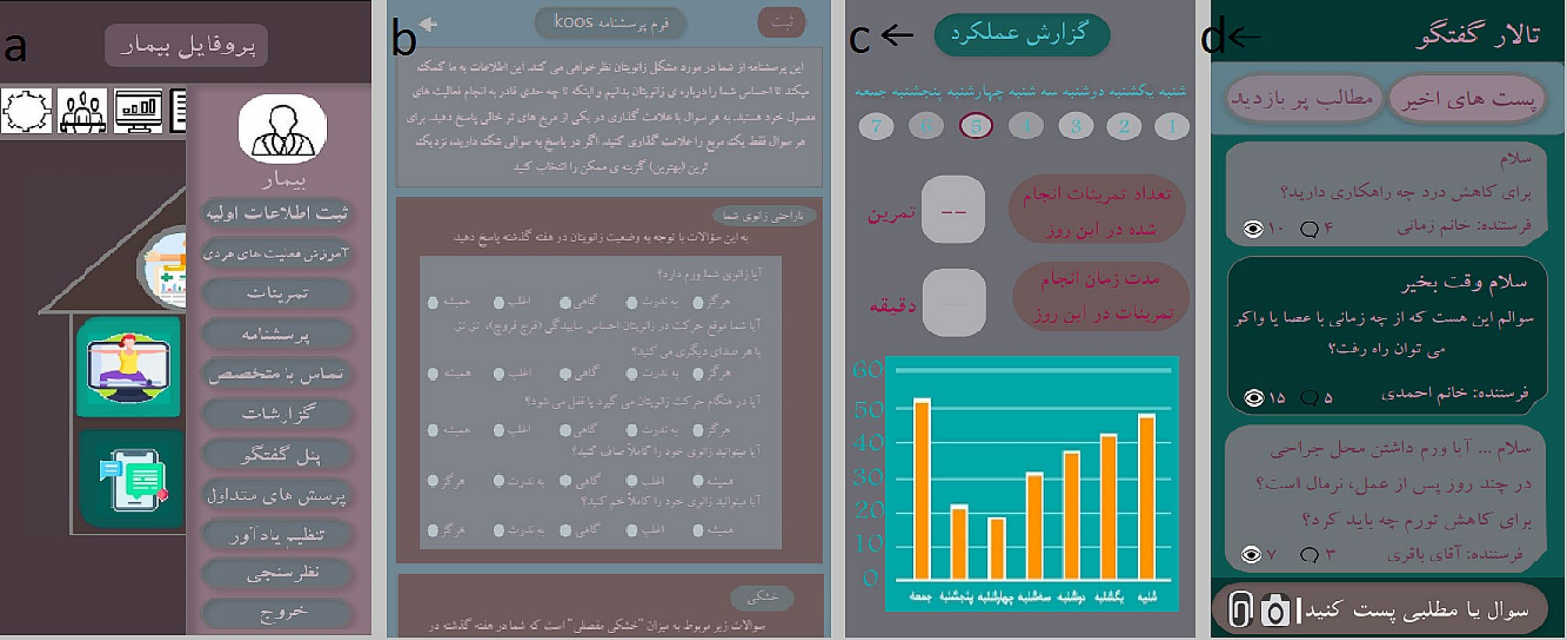
**a**: The menu of patient, 2-**b**: KOOS questionnaire, 2-**c**: Reports page, 2-**d**: Discussion panel

The home page of the patient user interface includes a menu to access other pages and app contents (it is shown in Fig. 2-a). This menu includes educational information (training on how to perform personal activities, frequently asked questions), exercises, forms (primary information form, KOOS questionnaire) [16, 25], reporting, communication with others (contact with an expert, discussion panel) and settings (setting a reminder, exiting the program).

#### 1. Page for teaching how to perform personal activities

On this page, there is educational information that includes information on how to perform daily activities in the first few weeks after surgery. Activities such as bathing, changing clothes, walking with crutches or a walker, going up and down the stairs, returning to work, and driving are taught in this section.

#### 2. Exercises page

**Fig. 3:**
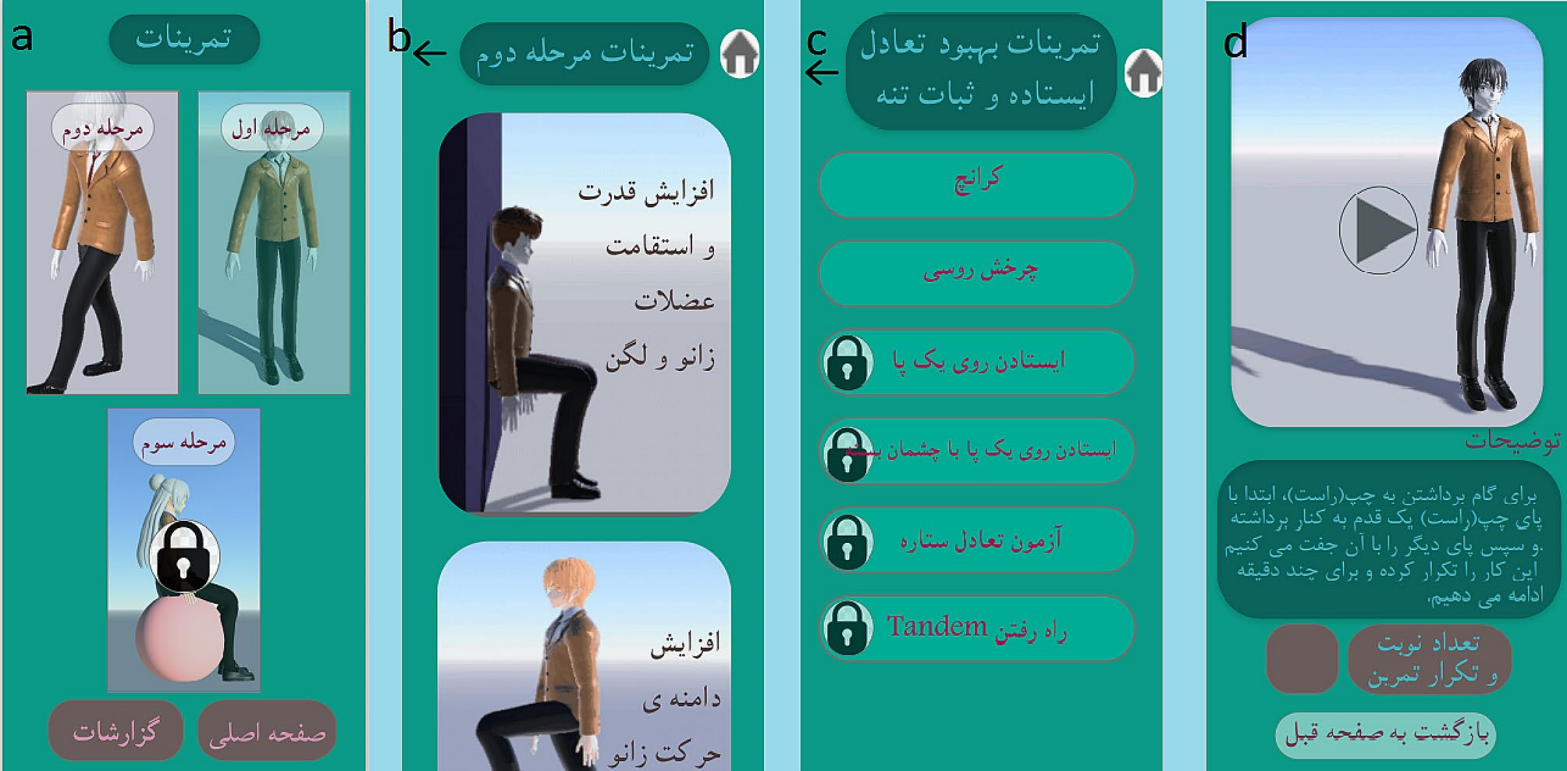
**a**: Exercises main page, 3-**b**: Exercises page of current stage, 3-**c**: list of exercises in current category, 3-**d**: final page of exercises

In the exercises section, the user can view the video and description of each exercise and perform the exercises. On the main page of the exercises (Fig. 3-a), there is a link to the exercises of all 3 steps, but the link to the exercises of the next steps is inactive for the user, and there is also a link to the previous steps, but this link is dim, which indicates that it has been skipped. By clicking on the link of the page, the user will be transferred to the page of the current stage; this page is shown in Fig. 3-b. On this page, the exercises of the current stage of the user are divided based on the purpose and application of the exercises. For example, the exercises of the second stage are divided into: 1-exercises to increase the strength and endurance of the knee and hip muscles, 2-exercises to increase the knee range of motion, 3-exercises to improve standing balance and trunk stability, and 4-exercises to improve functional movements.

By clicking on the link of each exercise category, the user can see the list of exercises in that category (Fig. 3-c). By clicking on the name of each exercise, the user will be transferred to the final page of the exercises and view a video of the exercise and the description (Fig. 3-d).

#### 3. KOOS questionnaire

The KOOS questionnaire was created as a tool to evaluate the patient’s opinion about the knee and related problems. This questionnaire is intended to be used in short-term time intervals, for example, to evaluate week-to-week changes due to treatment, as well as long-term time intervals. For this reason, the physician uses this questionnaire to measure the patient’s weekly changes and decide on the exercises that the patient should do in the next week and make these exercises available to the patient. You can see this *questionnaire* in Fig. 2-b.

#### 4. Contact with physician page

Users can chat with their physician through this page. It is also possible to make a voice or video call with the physician through this page.

#### 5. Reports page

In this section, users can see a graph of their activities in the last week, they can also choose a day from the calendar at the top of the page and see the report of this day, which includes the duration of the training and the number of exercises that the patient has done. You can see the reports page in Fig. 2-c.

#### 6. Discussion panel

Through this page, users can post questions and topics and share them with other users. Other users can comment on their opinions in the relevant section of this post. From the top of the conversation panel, you can choose one of the options of recent posts or the most visited content, the first option, sorts the posts by date and shows the newest posts first. The second option sorts the posts based on the number of visits to the post and shows the most visited posts first. You can see the discussion page in Fig. 2-d.

### Physician user interface

**Fig. 4:**
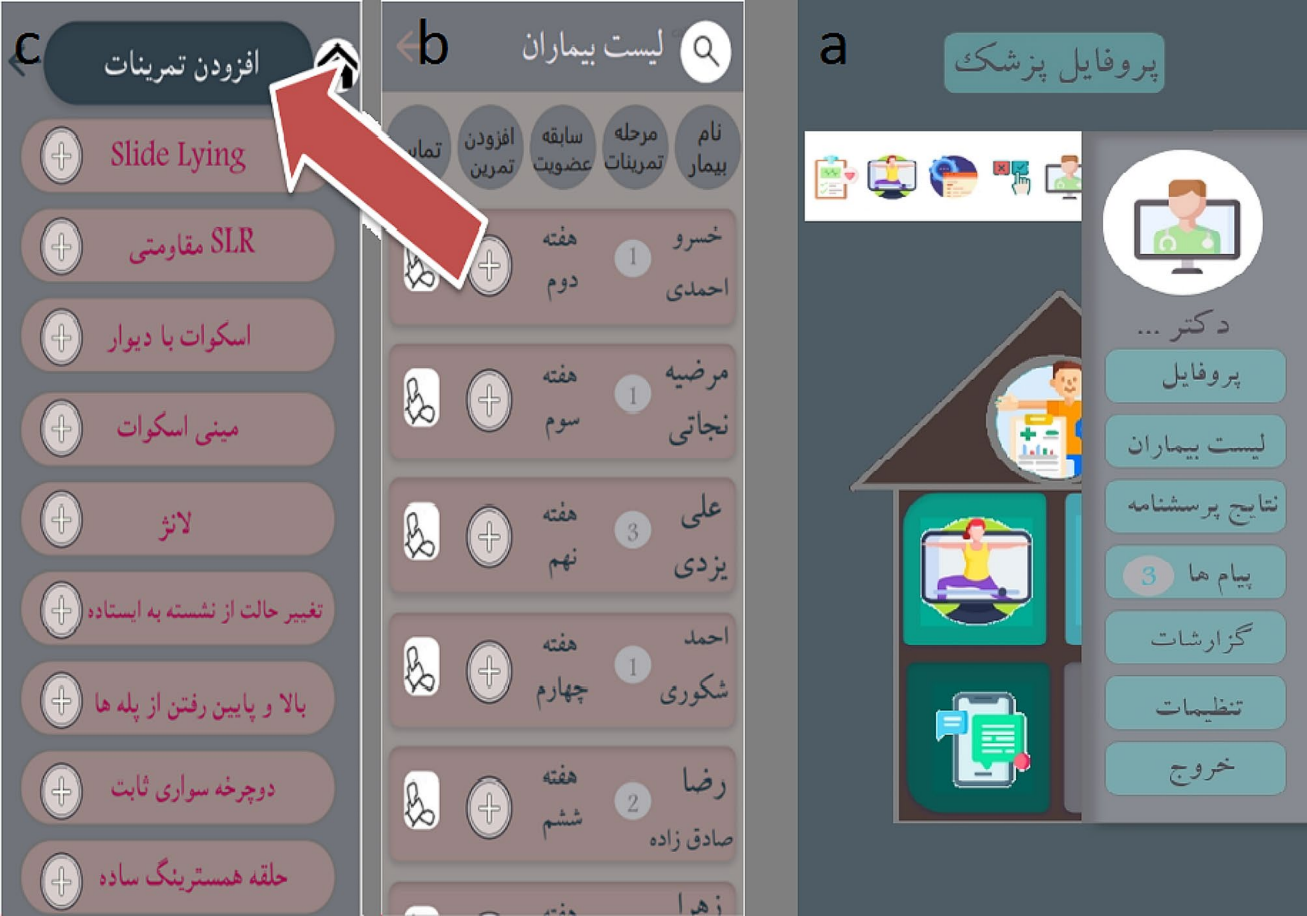
a: physician user interface, 4-b: List of patients, 4-c: add exercise to patient’s profile

In the main menu, the physicians can see their profile, patient list, results of the patients’ questionnaire, messages, reports, the settings page, and the exit page (Fig. 4-a).

#### 1. Physician’s profile

On this page, the physician can view and edit his information; the physician’s profile information is visible to other users.

#### 2. List of patients

In this section, there is a list of all patients, if the list is long; it is possible to search for the patient’s name. On this page, the physician can see the history of the patient’s membership and the stage of his exercises and can add to his exercises(Fig. 4-c) and also can contact the patient (Fig. 4-b).

### Step 4: system evaluation

After evaluating the usability by Nielsen’s Heuristic method, the strengths and weaknesses of the system were determined. The most common problems that were considered as weaknesses were related to the feature of helping users detect and recover errors, which can be solved with the technique of marking mandatory and optional data entry fields. Also, the degree of severity of the identified problems in the cases of the ability to view the system status, identification instead of a recall, and the ability to interact with other systems were at the lowest level (it was zero) and these are the strengths of the app. You can see the summary of the statistics related to usability evaluation in Table 3.

**Table 3:** Statistics of usability evaluation

Heuristic Evaluation Principles	Number of problems identified by evaluators			Total number of problems (%)	Average severity
	The first evaluator	The second evaluator	The third evaluator		
1. Clarity of system status	0	0	0	0 (0)	0
2. Consistency between the system and the real world	2	3	2	7 (11.8)	0.47
3. User control and freedom	0	1	0	1 (1.6)	0.09
4. Stability and standards	1	1	1	3 (5)	0.34
5. Error prevention	1	1	2	4 (6.7)	0.42
6. Recognizing instead of remembering	0	0	0	0 (0)	0
7. Flexibility and user efficiency	3	3	4	10 (16.9)	0.84
8. Beautiful and minimalist design	4	4	0	8 (13.5)	0.48
9. Identification, diagnosis and recovery of errors	3	3	2	8 (13.5)	0.09
10. Existence of documents	4	3	3	10 (16.9)	1.25
11. Proper interaction with other systems	0	0	0	0 (0)	0
12. Appropriate interaction with the user	1	1	0	2 (3.3)	0.25
13. privacy	2	2	2	6 (10.1)	1.84
Total	21	22	16	59 (100)	6.07

## Discussion

Knee problems cause difficulty in moving and doing daily tasks and affect the quality of life. Many patients with knee arthrosis need total knee replacement surgery. This surgery does not require a long-term stay in the hospital. However, on average complete recovery after this surgery, if they receive a suitable physiotherapy program, 6–12 months [7]. Therefore, proper rehabilitation activities are necessary to return to daily life after TKA. Still, the physiotherapy program imposes financial, time, and travel costs on patients, reducing adherence to the physiotherapy program. to reduce these costs, we use Telerehabilitation as an alternative to face-to-face visits.

The persian-language platform was not found to be suitable for Telerehabilitation and provides all the necessary features. Therefore we decided to present our Telerehabilitation program on the mobile application. Then, to design and develop the application, an interdisciplinary group including physiotherapy and medical informatics specialists came together to create an effective and acceptable application called KARA, and this expert-oriented application was developed.

The KARA Telerehabilitation self-care mobile app that we developed includes the ability of education, social support, increasing motivation, patient monitoring, reminders, Communication, reports, and also the ability to ask questions in the application. KARA application is different from previous apps in several ways: 1- Exercises are selected and personalized for the patient based on the physiotherapist’s opinion. In other apps that we observed, the exercises were fixed and predetermined, for example, in the App developed by other studies, the exercises were determined [17]. 2- Telerehabilitation has many benefits for patients and therapists, but the problem is that patients cannot manage their conditions remotely without having enough information [13]. In this App, there is basic information about how to perform activities after the operation and dos and don’ts in the short period after the surgery, in a separate section, which we did not see in similar apps. This section improves rehabilitation results by answering the patient’s questions and increasing their knowledge. 3- The correct way to perform therapeutic movements can be taught in the form of text or the form of animation and video. These trainings play a significant role in facilitating the rehabilitation process [38, 39]. The contents of this app are produced by the app developer and the contents are integrated and approved by the physician. For example, the image and video content are produced by the development team, which is not seen in many similar apps [17]. 4- Other available apps are suggested for help and must be accompanied by face-to-face physiotherapy sessions, but this app can be used alone for Telerehabilitation because it provides monitoring of the patient and changing the exercise program and continuous communication between the patient and the physiotherapist [17, 18, 20, 22–24, 26–30]. 5- Other apps are not so comprehensive and do not have all the features of this app, for example, some apps work well in the field of communication with the patient [17], while others offer a suitable exercise program or are suitable for monitoring the patient [17, 18, 20, 22–24, 26–30]. But this app has all these features and provides self-care, self-management, motivation, and reminder ability for patients.

We were facing some challenges during the development of the app.One of the challenges we faced was the preparation of text and video content for teaching exercises to patients. According to the opinion of the project consultant, the main sources were selected to prepare the content and were correctly translated into the language of the mobile application (Persian) under his supervision. The materials mentioned by the sources are the general type of exercises, and the details and names of the exercises of each stage were not clear, which were solved with the help of experts in this field.

To prepare video content, we checked different programs, and finally, we got help from the magic poser program, which allows us to make videos with anime style.

Another challenge was the issue of the application development costs, which was decided with the consensus of the research team to design and develop the prototype version of the program at this stage of the work [40].

This tool supports continuous communication between physiotherapists and patients. In fact, most of the self-care tools aim to help the physician and patients interact. Baker et al.‘s studies showed that mobile applications that provide communication between the patient and the care team provide better results for patients [13].

Another strength of the KARA app is the ability to share data and reports with healthcare providers. These reports can be in the form of graphs or textual data and include details of exercises performed by the patient and reports about performance indicators. This sharing helps the healthcare team to measure the patient’s progress and provide appropriate exercises for the next steps, which helps the patient to recover faster. The results of review studies on Telerehabilitation programs have shown that features such as sharing user experiences, communicating with experts, and reminding and viewing frequently asked questions of other patients are useful for helping users and this application has these features [17, 18, 20, 22–24, 26–30].

KARA application was also evaluated for usability, and this evaluation was conducted by Nielsen’s Heuristic principles, which showed promising results. But there were some problems, the biggest problem in the KARA application is related to the feature of helping users to detect and recover errors, which can be solved with the technique of marking mandatory and optional data entry fields, and no problems were found in the ability to view the system status, identification instead of reminders, and the ability to interact with other systems. By reviewing the evaluation results of similar apps, we found that some of these apps have major problems. For example, the results of Lyman’s study [13] showed that many users were not interested in this app, and there were also problems related to privacy concerns, which are serious concerns, the KARA app also have such problems, but to solve privacy concerns, it is necessary to consider several solutions: 1- Information storage must guarantee the security and integrity of data, which is also possible with the help of data encryption methods. 2- Information should be available to authorized people when they are needed based on the principle of access control. 3- Information should be stored securely in backup databases based on specific rules. 4- Information encryption protocols such as SSL certificates should be used to ensure the security of data being transmitted over public networks such as the Internet. We will implement this solutions for development of next versions of the app [41].

As a future work in the next steps we want to develop the commercial version of this app because a prototype has already been made. Then evaluate the effectiveness of the app. As a next step, we will expand this app to be suitable for preparation and use before knee replacement surgery. Support for other languages will also be considered.

## Conclusion

The Kara mobile app was designed and developed using an expert-centered process, for people who had total knee arthroplasty. The participation of users and other stakeholders in the design and development of health technologies is important because they determine the acceptability of the application. Access to information sources and appropriate exercises was identified as the main obstacle in the development of the application program. Kara App also showed successful results in the usability evaluation and can help facilitate the physiotherapy process in these patients and also improve treatment adherence and independence among these patients.

### Electronic supplementary material

Below is the link to the electronic supplementary material.

Supplementary Material 1

## Data Availability

All data generated or analyzed during this study are included in this published article.

## References

[1] Crowninshield RD, Rosenberg AG, Sporer SM. Changing demographics of patients with total joint replacement. Clin Orthop Relat Res. 2006;443:266–72. 16462450 10.1097/01.blo.0000188066.01833.4f

[2] Kahlenberg CA, et al. Patient satisfaction after total knee replacement: a systematic review. Hss j. 2018;14(2):192–201. 29983663 10.1007/s11420-018-9614-8 PMC6031540

[3] Wells VM, et al. Changing incidence of primary total hip arthroplasty and total knee arthroplasty for primary osteoarthritis. J Arthroplasty. 2002;17(3):267–73. 11938500 10.1054/arth.2002.30414

[4] Husted H, et al. Care principles at four fast-track arthroplasty departments in Denmark. Dan Med Bull. 2010;57(7):A4166. 20591341

[5] Kehlet H, Søballe K. Fast-track hip and knee replacement–what are the issues? Acta Orthop. 2010;81(3):271–2. 20446827 10.3109/17453674.2010.487237 PMC2876825

[6] Kehlet H. Fast-track hip and knee arthroplasty. Lancet. 2013;381(9878):1600–2. 23663938 10.1016/S0140-6736(13)61003-X

[7] Vehmeijer SBW, Husted H, Kehlet H. Outpatient total hip and knee arthroplasty. Acta Orthop. 2018;89(2):141–4. 29202644 10.1080/17453674.2017.1410958 PMC5901509

[8] Chan EY, et al. Postoperative pain following hospital discharge after knee replacement surgery: a patient survey. Pain Manag. 2013;3(3):177–88. 24654761 10.2217/pmt.13.14

[9] Chan EY, et al. Acute postoperative pain following hospital discharge after total knee arthroplasty. Osteoarthritis Cartilage. 2013;21(9):1257–63. 23973139 10.1016/j.joca.2013.06.011

[10] McDonall J, et al. Patient participation in postoperative care activities in patients undergoing total knee replacement surgery: Multimedia intervention for managing patient experience (MIME). Study protocol for a cluster randomised crossover trial. BMC Musculoskelet Disord. 2016;17:294. 27431239 10.1186/s12891-016-1133-5 PMC4950599

[11] Zhu NN, et al. Postoperative Pain Self-Management Behavior in patients who underwent total knee or hip arthroplasty. Aorn j. 2017;105(4):355–64. 28336024 10.1016/j.aorn.2017.02.001

[12] Kiani S, et al. Technical aspects of virtual augmented reality-based rehabilitation systems for musculoskeletal disorders of the lower limbs: a systematic review. BMC Musculoskelet Disord. 2023;24(1):4. 36597077 10.1186/s12891-022-06062-6 PMC9808732

[13] Brennan D, et al. A blueprint for telerehabilitation guidelines. Int J Telerehabil. 2010;2(2):31–4. 25945175 10.5195/ijt.2010.6063 PMC4296793

[14] Kisner CCL. B.J., *Therapeutic exercise: foundations and techniques*, ed. t. ed. 2017: Fa Davis.

[15] Nielson J. *10 usability heuristics for user Interface Design* updated Nov. Apr 24. 1994;15:2020.

[16] Z., H., Usability of tourism websites: a case study of heuristic evaluation. New Rev Hypermedia Multimedia, 2020. 26(1–2): p. 55–91.

[17] Stauber A, et al. RECOVER-E - a mobile app for patients undergoing total knee or hip replacement: study protocol. BMC Musculoskelet Disord. 2020;21(1):71. 32019529 10.1186/s12891-020-3090-2 PMC7001367

[18] Colomina J, et al. Implementing mhealth-enabled Integrated Care for Complex chronic patients with osteoarthritis undergoing primary hip or knee arthroplasty: prospective, Two-Arm, parallel trial. J Med Internet Res. 2021;23(9):e28320. 34473068 10.2196/28320 PMC8446839

[19] Ramkumar PN, et al. Artificial Intelligence and Arthroplasty at a single Institution: real-world applications of machine learning to Big Data, Value-Based Care, Mobile Health, and remote patient monitoring. J Arthroplasty. 2019;34(10):2204–9. 31280916 10.1016/j.arth.2019.06.018

[20] Jansson M, et al. The digital patient journey solution for patients undergoing elective hip and knee arthroplasty: protocol for a pragmatic randomized controlled trial. J Adv Nurs. 2020;76(6):1436–48. 32133684 10.1111/jan.14343

[21] Timmers T, et al. The Effect of an app for Day-to-Day Postoperative Care Education on patients with total knee replacement: Randomized Controlled Trial. JMIR Mhealth Uhealth. 2019;7(10):e15323. 31638594 10.2196/15323 PMC6914303

[22] de Batlle J, et al. Implementing Mobile Health-Enabled Integrated Care for Complex Chronic Patients: Patients and Professionals’ Acceptability Study. Volume 8. JMIR Mhealth Uhealth; 2020. p. e22136. 11. 10.2196/22136 PMC7718089 33216004

[23] Bäcker HC, et al. App-based rehabilitation program after total knee arthroplasty: a randomized controlled trial. Arch Orthop Trauma Surg. 2021;141(9):1575–82. 33547927 10.1007/s00402-021-03789-0 PMC8354977

[24] Hussain MS, et al. Supporting the delivery of total knee replacements care for both patients and their clinicians with a Mobile App and web-based Tool: Randomized Controlled Trial Protocol. Jmir Res Protocols. 2017;6(3):38–50. 10.2196/resprot.6498 PMC5352858 28249832

[25] Wang Y, et al. Patient-reported outcome measures used in patients undergoing total knee arthroplasty. Bone Joint Res. 2021;10(3):203–17. 33734821 10.1302/2046-3758.103.BJR-2020-0268.R1 PMC7998066

[26] Stauber A et al. RECOVER-E - A mobile app for patients undergoing total knee or hip replacement: study protocol. BMC Musculoskelet Disord, 2020. 21(1). 10.1186/s12891-020-3090-2 PMC7001367 32019529

[27] Hussain MS et al. Supporting the delivery of total knee replacements care for both patients and their clinicians with a mobile app and web-based tool: Randomized controlled trial protocol. JMIR Res Protocols, 2017. 6(3). 10.2196/resprot.6498 PMC5352858 28249832

[28] Wang Q et al. The effectiveness of internet-based telerehabilitation among patients after total joint arthroplasty: an integrative review. Int J Nurs Stud, 2021. 115. 10.1016/j.ijnurstu.2020.103845 33360248

[29] Correia FD, et al. Medium-term outcomes of Digital Versus Conventional Home-based Rehabilitation after total knee arthroplasty: prospective, parallel-group feasibility study. JMIR Rehabil Assist Technol. 2019;6(1):e13111. 30816849 10.2196/13111 PMC6416534

[30] Lyman S, et al. Monitoring patient recovery after THA or TKA using Mobile Technology. HSS J. 2020;16:358–65. 33380968 10.1007/s11420-019-09746-3 PMC7749883

[31] Borges PRT et al. Telerehabilitation program for older adults on a waiting list for physical therapy after hospital discharge: study protocol for a pragmatic randomized trial protocol. Trials, 2021. 22(1). 10.1186/s13063-021-05387-2 PMC8275917 34256830

[32] Msayib Y, et al. An Intelligent Remote Monitoring System for total knee arthroplasty patients. J Med Syst. 2017;41(6):90. 28421308 10.1007/s10916-017-0735-2

[33] Huang YP et al. Monitoring and assessment of rehabilitation progress on range of motion after total knee replacement by sensor-based system. Sens (Switzerland), 2020. 20(6). 10.3390/s20061703 PMC7147472 32197503

[34] Penders A et al. *SEKO: Smart system for assisting home-based rehabilitation of knee arthroplasty patients*. in *Proceedings – 2018 11th International Conference on Human System Interaction, HSI* 2018. 2018.

[35] Mateo KF. Effect of a smartphone app plus an accelerometer on physical activity and functional recovery during hospitalization after orthopedic surgery. J Clin Outcomes Manage. 2020;27(5):202–9. 10.12788/jcom.0024

[36] Reid H, et al. Patient and caregiver perspectives on an eHealth Tool: a qualitative investigation of Preferred Formats, features and characteristics of a presurgical eHealth Education Module. Rehabilitation Process and Outcome; 2021. p. 10. 10.1177/11795727211010501 PMC8282173 34497456

[37] Wang Q et al. Patients’ needs regarding rehabilitation services delivered via mobile applications after arthroplasty: A qualitative study. J Clin Nurs, 2021. 10.1111/jocn.16152 34859523

[38] Soever LJ, et al. Educational needs of patients undergoing total joint arthroplasty. Physiother Can. 2010;62(3):206–14. 21629598 10.3138/physio.62.3.206 PMC2909857

[39] Szöts K, et al. Physical health problems experienced in the early postoperative recovery period following total knee replacement. Int J Orthop Trauma Nurs. 2015;19(1):36–44. 25787815 10.1016/j.ijotn.2014.03.005

[40] Baniasadi T, et al. Study of challenges to utilise mobile-based health care monitoring systems: a descriptive literature review. J Telemed Telecare. 2018;24(10):661–8. 30343654 10.1177/1357633X18804747

[41] Zakerabasali S, Ayyoubzadeh SM. Internet of things and healthcare system: a systematic review of ethical issues. Health Sci Rep. 2022;5(6):e863. 36210869 10.1002/hsr2.863 PMC9528947

