# Supplementary material for: A home-based tele-rehabilitation exercise system for patients after knee replacement surgery
Source: BMC Musculoskelet Disord. 2024 Jul 31;25:605. doi: 10.1186/s12891-024-07731-4 (PMC11289961; doi:10.1186/s12891-024-07731-4)
Supplement: Supplementary file 1 — Supplementary Material 1 [file 12891_2024_7731_MOESM1_ESM.pdf]

Checklist of KARA application capabilities

The questionnaire presented to you is part of the medical informatics thesis project entitled "Development and evaluation of a remote rehabilitation system prototype for patients after knee replacement surgery" carried out at Shiraz University of Medical Sciences. The optimal design and implementation of this system facilitates remote rehabilitation for people who have undergone knee replacement (TKA).

Since the identification of the functional and non-functional capabilities of the system is one of the necessities of the design and development of this system, asking the experts in this field to finalize these capabilities is one of the very important and main stages of designing and creating a system. Undoubtedly, your valuable comments, as one of the experts and experts in this field, will help us in providing information and obtaining useful results, with the attention and precision you will give in answering the questions of the questionnaire.

Row	capability or content		completely agree	agree	No idea	disagree	completely disagree
1	socio-demographic	Gender					
2		Age					
3		Race					
4		BMI					
5		level of education					
6		working status					
7		Marital status					
8		Partner’s education					
9		Partner’s employment					
10		Number of pregnancies (for women)					
11		Alcohol use current					
12		Tobacco use current					
13	History of previous disease						
14	Ed	educate the patients with provide information on the disease					

Row	capability or content		completely agree	agree	No idea	disagree	completely disagree
15		Information for preparation to hospital stay					
16		Frequent monitoring with the tools					
17		Given information on rehabilitation, sports, sleep and returning to work					
18		Step-by-step tutorial training program					
19		Psychoeducation					
20		Relaxation techniques					
21		Yoga exercises/exercise guidelines					
22	social support	Social activity					
23		Interaction with other patient with TKA					
24		Support groups					
25	Motivation	Patients can set goals					
26		Interact with the app on a regular basis					
27		Congratulates them for special accomplishment					
28	Monitoring	Complete KOOS JR					
29		managing body weight and nutrition					
30		follow-up text message					
31		record symptoms and result of training					

Row	capability or content		completely agree	agree	No idea	disagree	completely disagree
32	reminder	Especial remind during phases when there is reduced contact with health care Professionals					
33	Communica tion	facilitate communication after discharge					
34		Therapist Interaction					
35		Feedback on physical activity					
36	system security	Security of patient data					
37		Confidentiality of patient data					
38		Authentication					
39	reports	Viewing moods/thoughts/activities graph for users and the care team					
40		Physician Report					
41		Patient Report					
42	interface User	Visibility of system status					
43		Match between system and the real world.					
44		User control and freedom to work with the application.					
45		Consistency and standards					
46		Error prevention					
47		Recognition rather than recall					
48		Flexibility and efficiency in use					

Row	capability or content		completely agree	agree	No idea	disagree	completely disagree
49	User interface	Aesthetic and minimalist design					
50		Help users recognize, diagnose, and recover from errors					
51		Help and documentation					
52		The application should be designed according to the skill level of the users					
53		Survey layout					
54		Personalization					
55		Enjoyability / Pleasurable and Respectful Interaction with the User					
56		Simplicity					
57		Usability					
58		The password and user information be editable					
59		Answers and questions					
